# Path analysis and empirical test of medical service enhancement for common prosperity under government participation

**DOI:** 10.3389/fpubh.2023.1076355

**Published:** 2023-03-14

**Authors:** Baoqi Chen, Xigang Zhang

**Affiliations:** School of Economics, Shandong University of Finance and Economics, Jinan, China

**Keywords:** medical services, medical fairness, medical efficiency, common prosperity, government participation, threshold effect

## Abstract

The level of medical services is the link between the two strategic goals of “healthy China” and “common prosperity,” and government participation plays an important adjustment role in it, so it is of great theoretical and practical significance to study its inner logic. In this paper, we firstly analyze the mechanism of medical service level to promote the development of common prosperity and the role of government in it; secondly, we construct a panel dynamic regression model and a threshold regression model to verify the relationship between the three. It is found that the contribution of both equity and efficiency of health care services to the achievement of common wealth is non-linear, and the degree of government participation plays an important adjustment role, with single and double threshold effects between them and the level of common prosperity, respectively. In the process of participating in the medical service market, the government should clearly position itself, actively play the demand-led role of the market, encourage private capital to provide quality medical services, and purposefully optimize the financial expenditure structure according to the local actual situation. There are many ways in which the government can be involved in health care, and there will be differences between China and other countries around the world. These are all worthy of further discussion.

## 1. Introduction and literature review

### 1.1. Introduction

The improvement of medical services is a key factor in improving the quality of the workforce, and since the 1980s the Chinese government has been committed to improving the medical conditions of its people through various policy instruments. The rapid economic development of China after the reform and opening up to become the second largest economy in the world has provided the material basis for the Chinese government to increase public healthcare services. The contradiction between the limited medical resources and the unlimited expansion of medical demand is becoming more and more prominent; the imbalance of medical resources allocation between urban and rural areas, regions and populations; local governments are keen on economic construction investment, but less enthusiastic about public services such as medical services, which are “large investment and slow to yield results,” and local governments are often “absent” in the medical service industry. Studies have shown that the improvement of medical services can promote the quality of regional economic development, and thus enhance the level of common prosperity, but the emergence of these problems has increased the uncertainty of this promotion effect. The dual attributes of the medical service industry and the characteristics of information asymmetry determine that the solution to these problems must rely on the government's macro-control ability.

At present, we still have a number of questions to answer: Does the improvement of medical service quality contribute significantly to the achievement of common prosperity, and what are its underlying mechanisms? Are the different aspects of medical service quality consistent in their contribution to common prosperity? How can the government play its role in the process of medical service development contributing to the realization of common prosperity?

### 1.2. Literature review

So far, many scholars have done a lot of research on three themes: medical services, government participation, and common prosperity. The improvement of the level of economic development is the proper meaning of common prosperity, and a large number of studies have shown that promoting the improvement of the level of health care services can promote economic development by improving the quality of human capital and driving the upgrading of industrial structure (1). Health expenditure has a significant effect on improving the quality of economic growth, with each 1% increase in health expenditure improving the quality of economic growth by about 0.06% (1, 2). There are at least three main interaction mechanisms between health and economic growth and economic development: first, health affects the production function through various direct or indirect channels; second, since health also affects people's utility levels, it affects physical capital investment and educational human capital investment in the form of health consumption and health investment; finally, economic growth and economic development can have an impact on health through the level and structure of food as well as nutrition consumption (2, 3) by using panel data from more than 100 countries, the development of the health investment sector was studied on the economic structure, and the results of the study showed that the development of health industry has a positive significance in promoting the transformation of industrial structure (3); in addition, the level of medical and health services has an impact on the well-being of residents' lives. Li and Chang (4) and Miao and Li (5) found that the adequacy, publicness and convenience of health care services have a significant impact on the happiness of rural residents' life by studying the relationship between health care services and the happiness of rural residents.

Although China's health care reform has been effective, a series of problems still exist in medical services (6). Based on the coefficient of variation, Thiel index, Gini coefficient and spatial autocorrelation model, found that the overall regional differences in the supply level of basic medical and health resources in China are large, but in recent years, they show a trend of fluctuation and narrowing (6, 7), the growth rate of total factor productivity in China's medical industry exists in significant differences between regions, showing the status quo of high in the west and low in the east (7, 8); by constructing a stochastic frontier production model, the study found that the overall efficiency level of China's health care system is low and there are significant regional differences (8, 9); there are different degrees of quantitative and qualitative imbalances in various types of health care resources in hospitals and primary health care institutions in China, and the greater the imbalance in the allocation of health care resources, the lower the efficiency of their utilization (9). Since the healthcare service industry has both commodity and public good attributes, the government has been playing an important role in its growth, but the results generated by this role are still controversial (10). After the government broke the pricing mechanism of drug prices affecting doctors' income, the supply side of medical services would obtain corresponding income through “excessive examination and equipment to support doctors,” and the price control of medical services would have little effect (10, 11); government spending and government competition can promote the improvement of medical and health services, but excessive government competition is inhibitory, and there are significant regional differences (11); government health spending “crowds out” residents' health care consumption, and the crowding-out effect decreases as residents' income increases (12).

Existing studies are important references for understanding the internal logic among the three, but they either study an issue relatively independently or are limited to discussing the mutual influence relationship between the two topics, and few scholars have conducted theoretical analysis and empirical tests on the internal logic among the three. Based on the results of the existing literature, this paper has the following marginal contributions: first, there are few studies in the existing literature on the path of medical service level improvement to promote the realization of common prosperity, and this paper adds in this aspect. Second, the paper argues whether the fairness and efficiency of medical service level are consistent in promoting common prosperity. Third, to address the uncertainty of government participation in medical services, the threshold effect between the level of medical services and the realization of common prosperity is verified using the degree of government participation as the threshold variable, and it is found that government participation within the threshold value can effectively promote the realization of common prosperity.

## 2. Analysis of the mechanisms of action

### 2.1. The path of medical service level improvement for common prosperity

The theoretical connotation of common prosperity is constantly enriched in practice, and the realization of common prosperity at different historical stages shows different characteristics. An accurate understanding of the contemporary characteristics of common prosperity is the prerequisite for a correct understanding of the influence of the level of medical services on the level of realization of common prosperity. The two basic elements of the common prosperity are “common” and “affluence”: “affluence” is expressed in the increase of the overall economic level of the country and the increase of individual disposable income. “Common” means the sharing of material wealth, which is expressed in the gradual reduction of regional differences, urban-rural differences and differences among people (13). Medical service is both an important component of national economic development and an important part of the equalization of basic public services, and has the dual attributes of economy and people's livelihood (14). Medical services can accelerate the realization of common prosperity in China by promoting the overall economic level and personal income level of the country and the people's sense of well-being.

#### 2.1.1. Macro level: Medical service development for economic scale increase

From the perspective of macroeconomic composition, consumption as one of the important driving force of macroeconomic growth, the drive for economic growth is increasingly apparent. China's large population, with the increase in per capita income and health awareness, people pay more attention to disease prevention, the number of medical institutions in China continues to grow, which in turn makes the rigid demand for medical industry grows, the medical economy consumption potential is constantly stimulated and accelerated release. The number of visits to hospitals and health institutions in China has increased from 5.83 billion in 2008 to 7.741 billion in 2020, and the total cost of medical treatment has increased from 200 billion yuan in 2010 to 722 billion yuan in 2020 (15).

From the perspective of the growth momentum of the macro-economy, with the rise of the new economic growth theory in the 1980s, new economists regarded technological progress and human capital as the main driving forces of economic growth (8, 16). Among many studies on human capital, education and health are the main factors determining the difference of human capital. The impact of the improvement of medical services on human capital includes both direct and indirect effects. First, the improvement of medical service level can directly improve workers' working years and work efficiency; second, the improvement of medical service level improves the return level of education investment in human capital investment by extending workers' working hours and life span, which further promotes the improvement of education investment and indirectly promotes the improvement of human capital. In the context of accelerating population aging and the gradual disappearance of demographic dividend, improving the quality of labor force will become a key driving force to promote economic development (17, 18).

#### 2.1.2. Industrial structure: The industrial restructuring effect of medical services

Medical service improvement boosts the development of health industry. In recent years, with the deepening of aging in China and the increasing attention of people to their own health problems, the health industry as a new service industry is gaining more and more attention and has gained rapid development (19). From the development status of health industry in developed countries, health industry has the characteristics of large investment scale, high rate of return and strong pulling effect, etc. With the transformation of China's economy from high speed to high quality development, the rapid development of the industry will become an important part of China's high quality economic development (20). Medical services as an important part of the health industry, the improvement of the level of medical services can indirectly promote the quality of economic development by promoting the development of the health industry (21).

Medical service development promotes the transformation of industrial structure. The improvement of the level of medical service industry attracts more capital to enter and also increases people's demand for health services and related products. The increase of capital investment and product demand will certainly attract more labor from primary and secondary industries to transfer to this industry, thus promoting the transformation and upgrading of industrial structure (22). In addition, with the improvement of technology, especially the development of digital technology, the boundaries of the health industry are gradually expanded, and the synergistic development effect between the health industry and other industries is enhanced, which further promotes the upgrading of the industrial structure. The optimization and upgrading of industrial structure will inevitably lead to the mutual transfer of industries in different regions, which can, to a certain extent, accelerate the industrial upgrading of backward regions.

#### 2.1.3. Micro aspect: Medical services increase the income of the population

While the level of consumption is an important factor affecting GNP from a macro perspective, the micro level of consumption depends on the income level of individual laborers. The impact of factors such as urbanization level, industrial structure optimization, and digital economy development on residents' income is exogenous, and the ability of residents to obtain income ultimately depends on individual working hours and skill level. The improvement of medical services can directly extend the working time and efficiency of individual workers, and indirectly promote the improvement of workers' skills. A general increase in the population's income can effectively reduce the income gap between populations (23, 24).

### 2.2. Ways of government participation in medical service level improvement

Medical services have different characteristics from general services and commodities, mainly in three aspects: firstly, medical services have the dual attributes of “public goods” and “commodities”; secondly, because of the strong professionalism of medical technology and the lack of medical knowledge of patients, there is a wide information asymmetry in the medical service market; finally, due to the government's entry regulation and the professionalism of medical technology, there is monopoly in the medical market. Finally, there is a monopoly in the medical service market due to the government's entry regulation and the professionalism of medical technology. The attributes and characteristics of medical services determine that there is a wide range of market failures in this field, which provides the necessary conditions for the government to intervene in the medical service market. Government participation in medical services in our economic practice mainly includes government subsidies and government regulation.

#### 2.2.1. Government subsidies

Government subsidies include both supply-side subsidies and demand-side subsidies. First, the supply-side subsidies can not only improve medical service technology and the supply of medical services, but also provide financial support to medical service providers, accelerate the renewal of medical equipment, promote medical technology, and promote the improvement of medical services (25). Second, demand-side subsidies can not only directly increase the demand-side medical consumption capacity, but also reduce consumers' preventive savings by changing their consumption expectations, thus increasing their current medical consumption level and promoting the development of the medical service industry. To a certain extent, government subsidies can promote the development of the health care industry, but excessive subsidies for health care services will inevitably crowd out funds invested in its public service projects or economic construction, and there is uncertainty about the impact on the overall economic development.

#### 2.2.2. Government regulation

The government regulation of medical service industry is mainly based on entry regulation and price regulation. In the process of implementing entry regulation in China, the entry threshold of medical institutions is controlled by setting the threshold of investment scale, qualification license and other measures. In the development of medical institutions, in the public medical field, each region is encouraged to focus on developing a certain number of public hospitals to improve the level of public welfare of medical services, and in the private medical field, social capital is encouraged to enter the medical service field to promote the development of medical institutions by introducing a competitive mechanism to force the improvement of the efficiency of medical institutions. In the implementation of price regulation, it is hoped that controlling pharmaceutical pricing will alleviate the long-standing problem of expensive medical care due to the imbalance between supply and demand of medical services in China. However, government regulation has its own shortcomings; the focused cultivation of public hospitals in the region will result in the monopoly phenomenon of medical institutions, and price regulation will weaken the enthusiasm of new drug research and development as well as the effective supply of drugs.

The theories related to government regulation mainly include public interest regulation theory and interest group regulation theory. According to the public interest regulation theory, the medical service market itself cannot form effective price constraints and mechanisms to limit the consumption of medical services due to its own defects, resulting in the inefficient allocation of resources in the field. The government is the protector of public interests, and through government regulation of the market, it can solve the market failure and thus optimize the allocation of resources and improve social welfare. However, the interest group regulation theory has a different attitude toward the role of government in the healthcare market. It believes that government regulation restricts the effective competition in the market and increases the interests of special interest groups, and advocates relaxing government regulation and relying on the efficiency of the free market to achieve efficiency optimization in the healthcare field. Based on the results of available empirical tests, the impact of government regulation on the market for health care services should be inconclusive. Different levels of government involvement and methods of involvement can have different effects on the improvement of medical services, and relevant evidence suggests that the effect of medical service quality on common wealth may be non-linear at different levels of government involvement.

### 2.3. The regulating role of government participation

There is no doubt that the improvement of medical services can contribute to the realization of common prosperity in China, but at the present stage, there are still many problems in the field of medical services in China. The basic characteristics of common prosperity are “common” and “prosperity,” and these two characteristics correspond to the field of medical services, which are expressed in the fairness and efficiency of medical services.

The fairness of medical services is reflected in two aspects of development: the accessibility and the availability. In terms of accessibility, it refers to the reasonable distribution of medical service institutions between urban and rural areas and between regions, and everyone can get the corresponding medical services within a reasonable space. Availability is mainly reflected in the price of medical services, which means that the consumption level of medical services is within the affordable range of the residents, and the high-income people and low-income people can get the most basic medical protection. The high efficiency of medical services can better promote the equity of medical services. The current contradiction between the rising demand of people for medical services and the insufficient supply and unbalanced allocation of medical resources is becoming more and more prominent. Limited to the current system and economic level, it is impossible to increase the supply of medical resources through a large-scale increase in investment in the short term, which makes it necessary for China to focus on the improvement of the efficiency of medical services. Due to the profit-driven mechanism of the market, it is impossible to achieve the fairness of medical services by purely relying on market forces, and the public goods attribute of medical services also determines that the market cannot solve its efficiency problems, so to achieve the fairness and efficiency of medical services, we must rely on the power of government macro-control (26, 27).

**Figure 1:**
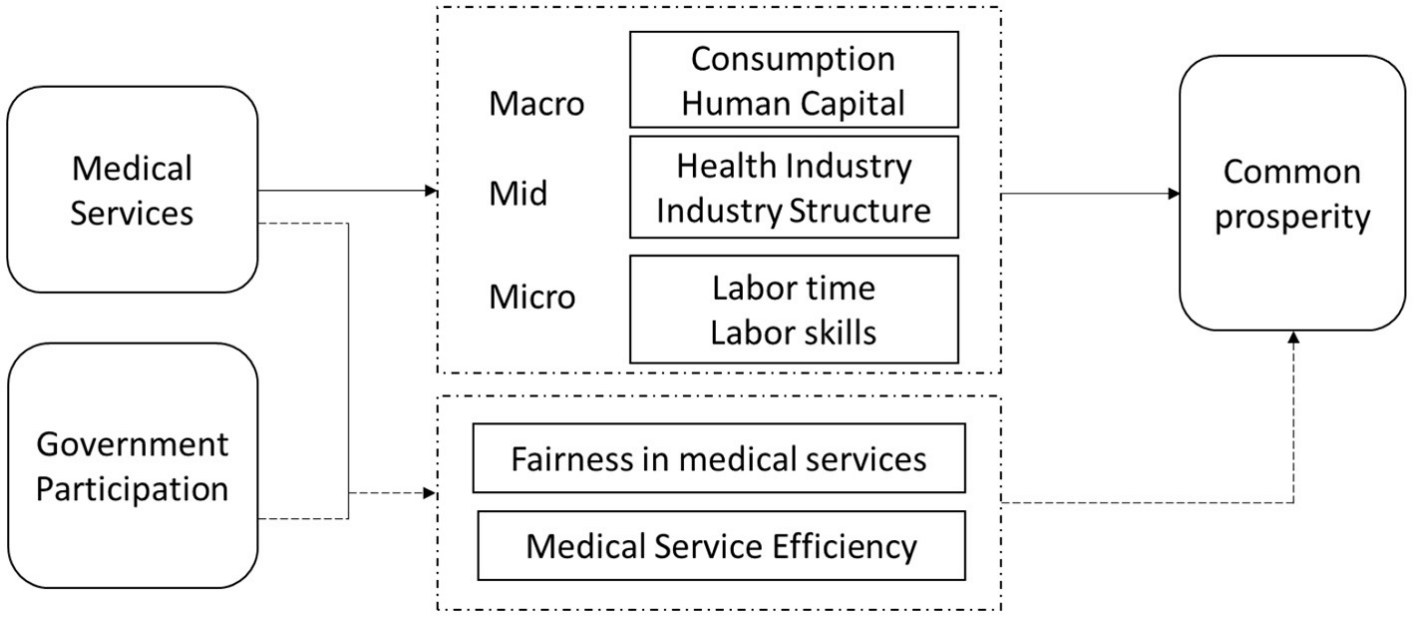
Research framework and transmission pathways for government participation, medical services, and the common prosperity.

The facts of China's medical services market also prove the need for government involvement. At present, China's medical service market is characterized by “induced consumption” and “excessive medical care” due to information asymmetry, and “difficulty in accessing medical care” due to uneven distribution of medical resources. This series of problems not only hinders the improvement of people's economic living standards and happiness, but also seriously hinders the realization of the goal of common prosperity in China. The solution of these problems requires government intervention, and the different degrees of government involvement and methods of participation will certainly have different results on the fairness and efficiency of medical services. The mechanism of action is analyzed as shown in Figure 1.

## 3. Variable descriptions and statistical characteristics

### 3.1. Variable description

#### 3.1.1. Explanatory variable: Level of achievement of common prosperity (Comp)

The measurement of the level of common prosperity involves both material and spiritual dimensions as well as “common” and “prosperous” aspects, so it cannot be measured by a single indicator. In this paper, based on relevant literature, we have constructed 2 primary indicators, 6 secondary indicators, 14 tertiary indicators and 27 bottom indicators according to the different characteristics of material and spiritual common prosperity (see Table 1), taking into account the availability of inter-provincial data. The evaluation results of the level of common prosperity achievement were calculated using the entropy weight method.

**Table 1:** Evaluation system of the level of common prosperity realization in China.

**Primary indicators**	**Secondary indicators**	**Tertiary indicators**	**Bottom indicators**	**Attribute**	**Weight**
Material common prosperity	Developmental	Economic benefits	Total labor productivity	+	0.0421
			GDP per capita	+	0.0371
			Disposable income per inhabitant	+	0.0392
		Economic structure	Resident consumption contribution rate	+	0.0097
			Engel coefficient	–	0.0119
			Foreign trade dependence	+	0.0841
	Shareability	Crowd gap	Gini coefficient	–	0.0318
		Urban-rural gap	Disposable income gap	–	0.0027
			Consumption gap	–	0.0066
			People's health care gap	–	0.0072
	Sustainability	Technology Innovation	R&D investment intensity	+	0.0699
			R&D personnel full time equivalent	+	0.1208
			Number of patents granted per 10,000 people	+	0.1037
		Capital accumulation	Labor accumulation	+	0.0091
			Human capital innovation power accumulation	+	0.0255
			Percentage of fixed asset investment	+	0.0219
		Ecological optimization	Emissions of major pollutants from exhaust gases	–	0.0089
			Emissions of major pollutants in wastewater	–	0.0113
			Growth rate of energy consumption per unit of GDP	–	0.0051
			Total afforestation area per 10,000 people	+	0.0834
Spiritual common prosperity	Personal enhancement	Culture and entertainment	Per capita expenditure on culture and entertainment	+	0.0359
		Library reading	Public library book and periodical literature lending attendance	+	0.0661
		Physical and mental health	Green space per capita	+	0.0153
	Cultural services	Industry support	Percentage of employees in the cultural and entertainment industry per 10,000 people	+	0.0996
		Government support	Culture and tourism business expenses accounted for the proportion of government financial expenditure	+	0.0265
	Social harmony	Family harmony	Divorce rate	–	0.0179
		Public safety	Number of traffic accidents	–	0.0065

The calculation method of some indicators is explained as follows: total labor productivity using the ratio of GDP to total employment in the three industries in each province; the contribution of residential consumption using the ratio of total retail sales of consumer goods to GDP in each province; foreign trade dependence using the share of imports and exports in GDP of each province; the urban-rural disposable income gap using the Thayer index of urban-rural income for each province; the urban-rural consumption gap using the ratio of urban per capita consumption expenditure to rural per capita consumption expenditure in each province; the urban-rural disparity in livelihood and health care uses the ratio of the number of health care beds per 1,000 people in urban areas to the number of health care beds per 1,000 people in rural areas in each province. R&D investment intensity using R&D expenditure as a proportion of GDP; human capital innovativeness accumulation using the number of students per 10,000 students in school. fixed asset investment ratio using the share of fixed asset investment in GDP; the ratio of the number of employees in the cultural and entertainment industry per 10,000 people is adopted as the ratio of the number of employees in the cultural and entertainment industry to the number of resident population in each province.

#### 3.1.2. Core explanatory variables

The government mainly influences the fairness and efficiency of medical services to make them better contribute to the realization of common wealth, so this paper sets two variables of fairness and efficiency of medical services as the core explanatory variables.

The fairness of medical services (Msf) is divided into two aspects: accessibility and availability. Accessibility is replaced by the number of medical beds available per capita. The number of medical beds per capita reflects, to a certain extent, the level of medical security and the distribution of resources for medical services, and a larger value indicates better accessibility of medical services. The ratio of per capita disposable income to per capita medical expenditure is used as an indicator of the availability of medical services, which can reflect the ability of residents' disposable income to pay for medical expenditure. The fairness of medical service is a comprehensive consideration of the availability and accessibility and both are positively related to the fairness of medical service, so we set the value of medical service fairness index as the product of availability and accessibility.

The efficiency of medical services (Mse) is mainly reflected in output efficiency, and this paper adopts the Malmquist index method proposed by Fare et al. in Data Envelopment Analysis (DEA) to measure the efficiency value of medical services, and learns from the research results of Yu (7), and the input indicators are selected as the number of health personnel, total assets of medical and health institutions, total medical and health costs, and the output indicators are selected as the number of consultations in health care institutions, and the number of discharges from health care institutions (7).

#### 3.1.3. Threshold variables

Government participation (Govpart) is influenced by the will of national and local governments in the direction of implementation, but the intensity of participation and the choice of participation in the implementation process are limited by the level of local fiscal revenue and economic development. For the research object of this paper, the ratio of local health care fiscal expenditure to local gross domestic product is used as a proxy variable for the degree of government participation.

#### 3.1.4. Control variables

In order to reduce the problem of endogeneity due to the omission of variables, this paper selects five factors as control variables, namely, the level of regional financial development (Fd), the degree of aging (Age), the level of education development (Edu), the degree of industrial structure optimization (Is), and the degree of industrialization (Ind). Among them, the level of regional financial development adopts the ratio of domestic and foreign currency loan balance to GDP of regional GDP as a proxy variable; the level of regional aging adopts the ratio of regional elderly dependency as a proxy variable; the level of industrial structure optimization adopts the ratio of output value of tertiary industry to GDP of regional GDP as a proxy variable; the level of industrialization adopts the ratio of industrial value added to regional GDP.

### 3.2. Data sources and statistical characteristics

#### 3.2.1. Data sources

Due to the lack of relevant data for Hong Kong, Macao and Taiwan in China, and the lack of data for 2009 and 2010 for some provinces. For 2020, the outbreak of COVID-19 virus caused an abnormal increase in the level of medical consumption, therefore, this paper selects panel data from 2010 to 2019 for 31 provinces (autonomous regions and municipalities directly under the central government) in China. The data are obtained from the China Statistical Yearbook, China Statistical Yearbook of Culture and Related Industries, local government work reports and statistical yearbooks of each province, and the missing data for some years in individual provinces are extrapolated from the rate of change of nearby years.

#### 3.2.2. Statistical characteristics of data

lnMsfGovpart indicates the interaction item of medical service fairness and government involvement; MseGovpart indicates that the efficiency of medical services and government involvement in the interaction items; lnMsf2 denotes the quadratic term of lnMsf; Mse2 denotes the quadratic term of Mse. The statistical description of the various data is shown in Table 2.

**Table 2:** Statistical description of data for each variable.

**Variables**	**Max**	**Min**	**Mean**	**Std**
Comp	0.523	0.104	0.229	0.084
lnMsf	7.215	3.238	5.693	0.876
Mse	1.171	0.559	0.959	0.077
Govpart	0.073	0.006	0.019	0.010
Fd	2.783	0.669	1.436	0.452
Age	0.237	0.067	0.139	0.029
Edu	0.219	0.099	0.160	0.025
Is	0.836	0.286	0.465	0.095
Ind	0.748	0.067	0.374	0.131
lnMsfGovpart	0.312	0.04	0.113	0.046
MseGovpart	3.47	0.038	0.525	0.62
lnMsf2	52.056	10.483	33.167	8.411
Mse2	1.371	0.312	0.924	0.140

## 4. Model construction and analysis of results

### 4.1. Dynamic panel model construction and result analysis

#### 4.1.1. Description of estimation method and model form

Because common prosperity is a dynamic development process, the achievement of each milestone is bound to be influenced by the development results of the previous phase, so it is necessary to include the lagged term of the common prosperity achievement level index as an explanatory variable in the process of constructing the model; in addition, considering the possible endogeneity problem among the variables, combined with the characteristics of balanced panel data, this paper learns from Arellano and Bond (15) and Blundell and Bond's Generalized Method of Moments (GMM) for the econometric analysis (28). Compared with the differential GMM, the systematic GMM can solve the problem of weak instrumental variables that may exist in the differential equation, and can use the information of variable level changes and differential changes to effectively overcome the bias caused by the inclusion of lagged terms of the explained variables in the explanatory variables, and solve the endogeneity problem caused by a part of omitted variables that change over time but not with individuals (29–31).

Based on the previous mechanism analysis, the following dynamic panel model is constructed to systematically analyze the following three questions: are the fairness and efficiency of medical services important factors affecting the achievement of common prosperity? Is the impact of medical service level on common affluence non-linear? Does the level of government involvement play a moderating role in the relationship between the level of health care services and common prosperity?

A dynamic panel regression model of the form of equation (1) is constructed to analyze whether the improvement of medical services can contribute to the achievement of common prosperity.


(1) $$\begin{array}{l} Comp_{it}=\alpha_{0}+\alpha_{1}Comp_{it-1}+\alpha_{2}\ln\;Msf_{it}+\alpha_{3}Mse_{it}+\varepsilon_{it} \end{array}$$

(2) $$\begin{array}{l} Comp_{it}=\beta_{0}+\beta_{1}Comp_{it-1}+\beta_{2}\ln\;Msf_{it}+\beta_{3}Mse_{it}\\ \;+\beta_{4}Govpart_{it}+\varepsilon_{it} \end{array}$$

(3) $$\begin{array}{l} Comp_{it}=\lambda_{0}+\lambda_{1}Comp_{it-1}+\lambda_{2}\ln\;Msf_{it}+\lambda_{3}Mse_{it}\\ \;+\lambda_{4}Govpart_{it}+\lambda_{5}X_{it}+\varepsilon_{it} \end{array}$$

Equation (2) is to add the variable of the degree of government participation to Equation (1) to investigate the role of the degree of government participation in the process of increasing the level of common prosperity achievement. Equation (3) is based on Equation (2) by adding control variables that may have an impact on the common prosperity index in order to test the reliability of the results of Equation (2).

The above model can only verify whether the level of medical service and government participation have any effect on the level of achieving common prosperity, and whether there is a non-linear relationship between them needs further model construction and analysis. Equations (4) and (5) is based on Equation (3) and introduces the quadratic term of medical service level as the independent variable to verify whether the relationship between the two is non-linear. If the non-linear relationship exists, whether the degree of government participation is the cause of this relationship needs to be further demonstrated. Equations (6) and (7) are based on Equation (3) by adding the cross term of the level of medical service and the degree of government participation to investigate the moderating role of the degree of government participation in the process of medical service level contributing to the achievement of common prosperity.


(4) $$\begin{array}{l} Comp_{it}=c+X_{1}Comp_{it-1}+X_{2}\ln\;Msf_{it}+X_{3}Mse_{it}\\ \;+X_{4}Govpart_{it}+X_{5}\ln\;Msf_{2}it+X_{6}X_{it}+\varepsilon_{it} \end{array}$$

(5) $$\begin{array}{l} Comp_{it}=c+\iota_{1}Comp_{it-1}+\iota_{2}\ln\;Msf_{it}+\iota_{3}Mse_{it}+\iota_{4}Govpart_{it}\\ \;+\iota_{5}\ln\;Msf_{2}it+\iota_{6}\iota_{it}+\varepsilon_{it} \end{array}$$

(6) $$\begin{array}{l} Comp_{it}=c+\kappa_{1}Comp_{it-1}+\kappa_{2}\ln\;Msf_{it}+\kappa_{3}Mse_{it}\\ \;+\kappa_{4}Govpart_{it}+\kappa_{5}\ln\;Msf_{2}it+\kappa_{6}\kappa_{it}+\varepsilon_{it} \end{array}$$

(7) $$\begin{array}{l} Comp_{it}=c+\upsilon_{1}Comp_{it-1}+\upsilon_{2}\ln\;Msf_{it}+\upsilon_{3}Mse_{it}\\ \;+\upsilon_{4}Govpart_{it}+\upsilon_{5}\ln\;Msf_{2}it+\upsilon_{6}\upsilon_{it}+\varepsilon_{it} \end{array}$$

Where: are the coefficients of the explanatory variables, ε are the error terms of the corresponding models, *c* is a constant term, and *X* is some series of control variables.

#### 4.1.2. Analysis of model estimation results

Systematic GMM estimation was performed for the above models, and the AR (2) statistics for all models regression results were greater than 0.05, indicating that there was no second-order autocorrelation, and the Hansen test values for each model ranged from 0.1 to 0.25, indicating that the instrumental variables selected in the models were valid and there was no over-identification problem. The specific regression results for each model are shown in Table 3.

**Table 3:** Systematic GMM regression results for Equations 1–7.

	**(1)**	**(2)**	**(3)**	**(4)**	**(5)**	**(6)**	**(7)**
L. comp	0.917^***^	0.879^***^	0.674^***^	0.852^***^	0.725^***^	0.756^***^	0.531^***^
	(0.06)	(0.024)	(0.132)	(0.068)	(0.052)	(0.035)	(0.145)
lnmsf	0.027^***^	0.019^***^	0.026^***^	0.065^**^	0.039^**^	0.031^***^	0.058^**^
	(0.005)	(0.006)	(0.023)	(0.032)	(0.025)	(0.017)	(0.04)
Mse	0.065^***^	0.083^**^	0.048^**^	0.091^*^	0.059^***^	0.049^**^	0.112^***^
	(0.013)	(0.035)	(0.031)	(0.621)	(0.024)	(0.03)	(0.042)
Govpart		0.687^**^	0.564^***^	0.647^**^	0.652^**^	0.549^**^	0.648^***^
		(0.275)	(0.779)	(0.583)	(0.257)	(0.841)	(0.751)
Mse2				−0.586^**^			
				(0.248)			
lnMsf2					0.014		
					(0.075)		
lnMsfGovpart						0.498^***^	
						(0.71)	
MseGovpart							0.315^**^
							(0.042)
Fd			−0.012	0.031^**^	0.042^***^	0.026^**^	0.024^*^
			(0.036)	(0.045)	(0.024)	(0.017)	(0.021)
Age			−0.223	−0.325^**^	−0.301^***^	−0.036	−0.613^**^
			(0.167)	(0.033)	(0.094)	(0.049)	(0.227)
Edu			0.150^*^	0.313	0.045	0.046^*^	0.418
			(0.212)	(0.324)	(0.145)	(0.126)	(0.269)
Is			0.525^***^	−0.004	0.142^***^	0.076^**^	0.464^**^
			(0.203)	(0.086)	(0.037)	(0.028)	(0.214)
Ind			0.423^**^	0.324	0.165^*^	0.016	0.29^**^
			(0.195)	(0.047)	(0.034)	(0.015)	(0.118)
cons	−0.202^***^	−0.198^***^	−0.739^***^	0.124^***^	−0.012^***^	−0.157^***^	−0.642^***^
	(0.048)	(0.12)	(0.167)	(0.291)	(0.058)	(0.053)	(0.157)
AR(2)	0.387	0.424	0.353	0.189	0.276	0.254	0.463
Hansen test	0.124	0.225	0.152	0.227	0.191	0.268	0.126

(1) ^***^, ^**^, ^*^ denote significant at the 1%, 5%, and 10% statistical levels. (2) L. comp is the first-order lagged term of the common prosperity index. (3) The values in parentheses are robust standard errors.

In general, the regression results of all seven equations show that the first-order lagged term of the common prosperity index is significant at the 1% statistical level and the regression coefficients are all positive, indicating that there is a significant time continuity in the improvement of the level of common prosperity realization in China, and the realization of common prosperity in the early period can provide positive promotion for the later period. From the comparison among the equations, we can see that both the fairness and efficiency of medical services are important factors affecting the realization of common wealth through the estimation results of Equation (1), and from the coefficient size, the current efficiency of medical services has a more obvious role in promoting common wealth; Equation (2) increases the variable of the degree of government participation, although the coefficient of fairness of medical services decreases from Although the coefficient of fairness of medical service decreases from 0.027 to 0.019 and the coefficient of efficiency of medical service increases from 0.065 to 0.083, the significance level of both does not change fundamentally, and the regression result of Equation (2) shows that the size of government participation is also one of the factors that affect the realization of common prosperity in China; from the regression result of Equation (3), the addition of control variables does not substantially change the results of Equations (1) and (2), which further indicates that the conclusion obtained from Equations (1, 2) is stable and reliable.

Equations (4)–(7) are constructed mainly to analyze whether there is a non-linear relationship between the level of medical services and common prosperity. According to the regression results of Equation (4), the coefficient of the quadratic term of the efficiency of medical services is negative and significant at the 5% statistical level. Thus, it can be judged that there is an inverted U-shaped non-linear relationship between the level of common prosperity and the efficiency of medical services in China. This means that before the inflection point, the efficiency of medical services can promote the level of common prosperity, but after the inflection point, the efficiency of medical services can hinder the achievement of common prosperity. This is mainly because, under the condition of limited resources, the rapid increase of medical service efficiency will cause the loss of equity to a certain extent, and the efficiency increase will hinder the process of achieving common prosperity when the promotion effect of efficiency increase is smaller than the hindrance effect of equity loss, on the contrary, the efficiency increase can promote the achievement of common prosperity. The regression results of Equation (5) do not indicate that there is a significant relationship between the quadratic term of the level of common prosperity and the fairness of medical services, so we can judge that there is no U type relationship between the two, but whether there is other types of non-linear relationship needs further investigation. The regression results of Equations (6) and (7) show that the coefficients of the interaction term between government participation and fairness and efficiency of health care services are positive and significant at least at the 5% level, indicating that government participation plays a moderating role in the improvement of medical services and the achievement of common prosperity. The coefficient of the interaction term between government participation and medical fairness is larger than the coefficient of the interaction term between government participation and medical efficiency, which indicates that government participation plays a more significant role in regulating the fairness of medical services to promote the achievement of common prosperity. In conclusion, the contribution of the level of medical service to the realization of common prosperity in China will be influenced by the degree of government participation, and the size and timing of government participation need to be further analyzed through the threshold effect.

#### 4.1.3. Curves based on LOWESS method

**Figure 2:**
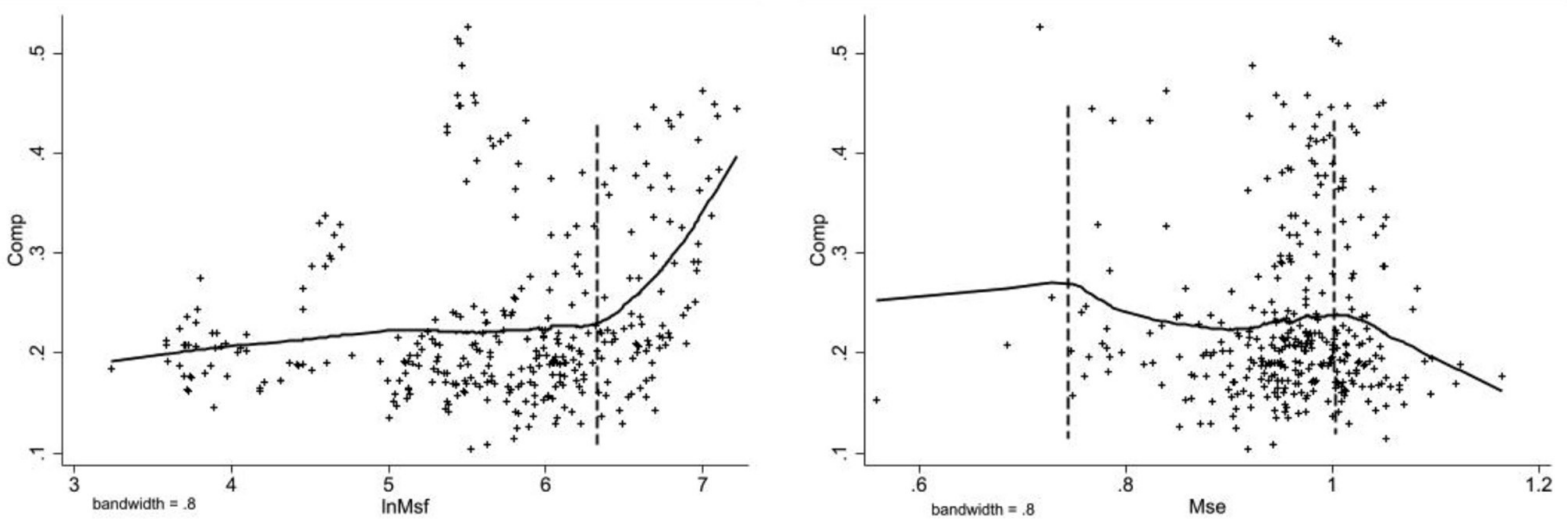
LOWESS curve.

In order to further verify the existence of non-linear relationships between variables from the perspective of data, the LOWESS method is applied to curve-fit the equity and efficiency of medical services and the level of achievement of common wealth, respectively. The LOWESS method is a non-parametric fitting method that is not constrained by the overall distribution and parameters, and can be used to do preliminary if the relationship or functional form between variables is uncertain analysis. The result is shown in Figure 2.

First, the fairness of medical service can promote the improvement of the level of common prosperity, and there are obvious stages between them. Secondly, the effect of efficiency of medical services on common prosperity can be divided into three intervals, and the overall relationship shows “an inverted U-shaped” non-linear relationship. The regression results of the dynamic panel model show that both fairness and efficiency of medical services are important factors affecting the level of common wealth. Different levels of government participation will have different moderating effects on both, and the degree and timing of government participation need to be further analyzed.

### 4.2. Threshold regression model construction and result analysis

#### 4.2.1. Threshold regression model construction

According to the previous mechanism analysis and the regression results of the dynamic panel model, both aspects of equity and efficiency of medical services can contribute to the improvement of the level of common prosperity achievement, but the different degrees of government participation in the process will have different effects on the relationship between the two. In view of this, a double-threshold panel model of the form of Equations (8, 9) is developed by learning Hansen's (32) threshold regression method and using the degree of government involvement as the threshold variable.


(8) $$\begin{array}{l} {Comp}_{it}=c+\tau_{1}\ln\;Msf_{it}I\left(Govpart_{it}\leq \gamma_{1}\right)\\ \;+\tau_{2}\ln\;Msf_{it}I\left(\gamma_{1}<Govpart_{it}\leq \gamma_{2}\right)\\ \;+\tau_{3}\ln\;Msf_{it}I\left(Govpart_{it}>\gamma_{2}\right)+\tau_{4}Mse_{it}\\ \;+\tau_{5}X_{it}+\varepsilon_{it} \end{array}$$

(9) $$\begin{array}{l} {Comp}_{it}=c+\rho_{1}Mse_{it}I\left(Govpart_{it}\leq \Omega_{1}\right)\\ \;+\rho_{2}Mse_{it}I\left(\Omega_{1}<Govpart_{it}\leq \Omega_{2}\right)\\ \;+\rho_{3}Mse_{it}I\left(Govpart_{it}>\Omega_{2}\right)+\rho_{4}\ln\;Msf_{it}\\ \;+\rho_{5}X_{it}+\varepsilon_{it} \end{array}$$

In the equation: τρ are model coefficients, γΩ are threshold values, *c* is the constant term, ε is the error term, and *I*(·)is the indicator function. The construction, parameter estimation and testing process of the single-threshold panel model and the three-threshold panel model are similar to those of the double-threshold panel model, and are not repeated in this paper.

**Figure 3:**
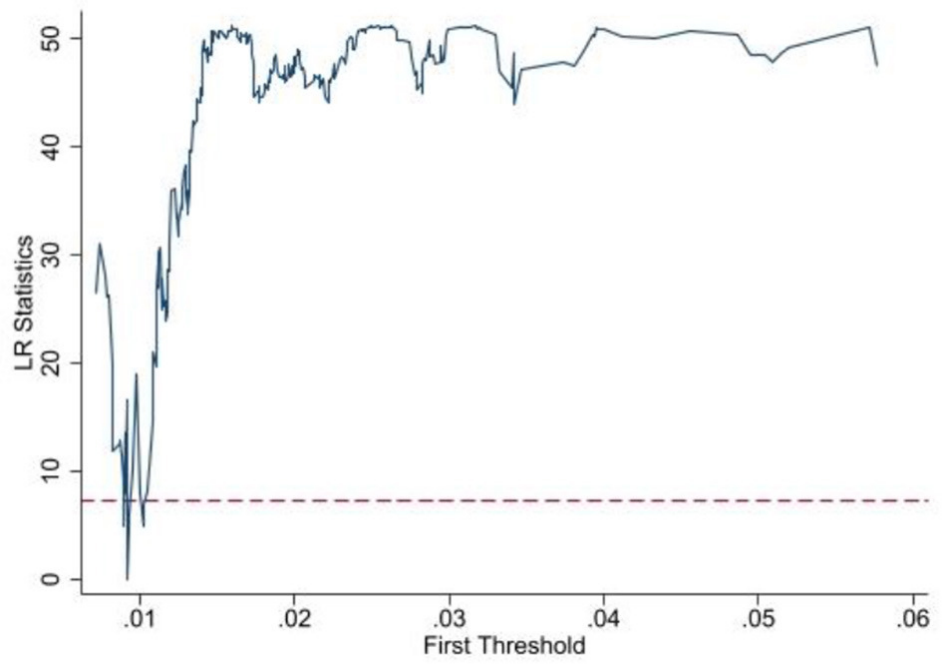
LR statistics chart on lnMsf.

**Figure 4:**
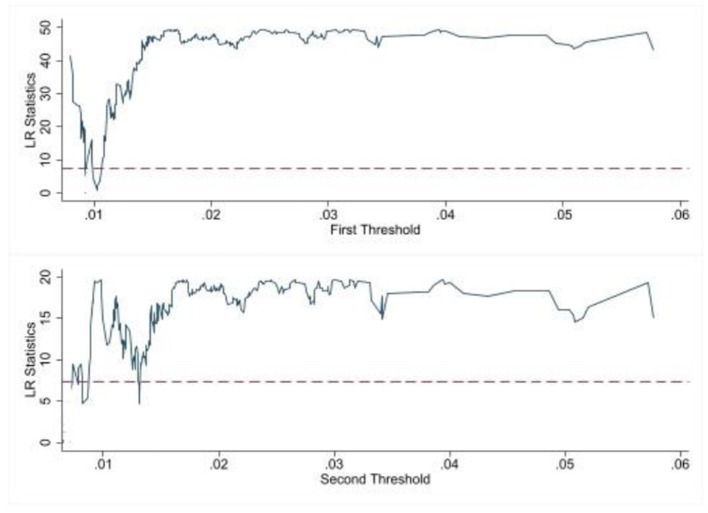
LR statistics chart on Mse.

Before parameter estimation, the number of threshold values and significance of the model are first tested to determine the specific form of the model in this paper. In this paper, the asymptotic value of the F-statistic is obtained by the threshold effect bootstrap sampling (Bootstrap) test, and the *p*-value is obtained, and the threshold is tested at 400 grid searches as well as 300 bootstrap samples, respectively. According to the test results, there is a single threshold effect for the fairness of medical services when the degree of government participation is used as the threshold variable, and the threshold value is 0.9%, and there is a double threshold effect for the efficiency of medical services, and the threshold values are 1.1% and 1.4%, respectively. The results of the tests are shown in Table 4 and Figures 3, 4.

**Table 4:** Bootstrap test results.

	**lnMsf**		**Mse**	
**Threshold**	**Fstat**	**Prob**	**Fstat**	**Prob**
Single	49.670	0.000	54.370	0.000
Double	20.350	0.230	21.350	0.018
Triple	12.560	0.670	15.880	0.562

#### 4.2.3. Analysis of threshold regression results

According to the results of the threshold effect test, different types of threshold models were used to estimate the threshold effects, and the results are shown in Table 5. The coefficient of fairness of medical service is always positive in line with the estimated results of dynamic panel model, which indicates that the improvement of fairness of medical service will always promote the level of common prosperity achievement. The coefficient of medical service efficiency changes from positive to negative with the change of the threshold variable, which verifies the inverse “U” non-linear relationship between medical service efficiency and the level of common prosperity in the system GMM model, and indicates that the degree of government participation plays an important moderating role in the process of medical service efficiency for common prosperity.

**Table 5:** Threshold regression results.

	**(8)**	**(9)**
lnMsf (govpart ≤ 0.009)	0.042^***^	
	(0.000)	
lnMsf (govpart > 0.009)	0.049^***^	
	(0.000)	
Mse (govpart ≤ 0.011)		0.028^***^
		(0.002)
Mse (0.011 < govpart ≤ 0.014)		0.056^***^
		(0.000)
Mse (govpart > 0.014)		−0.008^**^
		(0.049)
lnMsf		0.043^***^
		(0.000)
Mse	0.027^**^	
	(0.035)	
Fd	0.006	0.007^*^
	(0.167)	(0.063)
Age	0.364^***^	0.338^***^
	(0.000)	(0.000)
Edu	0.134^*^	0.078
	(0.068)	(0.377)
Is	0.275^***^	0.271^***^
	(0.000)	(0.000)
Ind	0.054^**^	0.056^**^
	(0.021)	(0.028)
cons	−0.283^***^	−0.248^***^
	(0.000)	(0.000)

^***^, ^**^, ^*^ Significant at 1%, 5% and 10% statistical levels, F-statistic and p-value (in parentheses) obtained from 300 Bootstrap self-samplings.

From the regression results of Equation (8) in Table 5, it is clear that there is a significant threshold effect between the fairness of medical services and the level of common prosperity achievement, when the degree of government participation is < 0.9%, the fairness of medical services and the level of common prosperity achievement are significantly positively correlated at the 1% level, with a coefficient of 0.042; when the degree of government participation exceeds 0.9%, the level of medical services has a significantly stronger contribution to the increase of the level of common prosperity achievement The result of Equation (8) indicates that the fairness of medical services is non-linearly positively correlated with the achievement of common prosperity, i.e., the stronger the fairness of medical services, the greater the effect on the enhancement of the achievement level of common prosperity.

The regression results of model (9) show that the relationship between medical service efficiency and common prosperity under government participation shows obvious multi-stage characteristics. When the degree of government participation is below 1.1%, the coefficient of medical service efficiency is 0.028, and at 1% significance level, the increase of common prosperity for each 1% increase in medical service efficiency is 0.029%; when the degree of government participation is between 1.1% and 1.4%, the coefficient of medical service efficiency becomes 0.056, and the promotion effect on common prosperity is significantly increased at 1% significance level; when the degree of government participation is over 1.4%, the coefficient of medical service efficiency changes from positive to positive. The coefficient of medical service efficiency changes from positive to negative at the 5% level of significance when the degree of government participation exceeds 1.4%. The results of model (9) show that the relationship between medical service efficiency and the achievement of common prosperity is in an inverted “U” shape, and the contribution of medical service efficiency to common prosperity increases gradually with the increase of government participation before the inflection point, but after the inflection point, the contribution of medical service efficiency to common prosperity The “shift” occurs after the inflection point. This “shift” phenomenon may be due to the limited resources, i.e., the increase of government expenditure on medical services has weakened the development of other basic public services; it may also be due to the increase of medical service efficiency without taking into account the fairness of medical services, resulting in the reduction of the overall level of medical services.

## 5. Research conclusions and policy recommendations

Health for all is an important support for the strategy of “Healthy China” and a prerequisite for achieving the goal of common prosperity, in which the high level of development of medical service industry plays a key role. Based on this, this paper firstly makes the necessary theoretical analysis on the path of improving medical services to promote the realization of common prosperity; secondly, it constructs a systematic GMM model with the fairness and efficiency of medical services as the core explanatory variables and a panel threshold regression model with the degree of government participation as the threshold variable, respectively.

The study found that:

First, in general, the improvement of medical service level has a significant contribution to the realization of common prosperity, and the efficiency of medical service has a more significant contribution to common prosperity. The government is both a market manager and a market participant, and government participation itself has a significant positive effect on the realization of common prosperity as well as the level of medical service development.

Second, the equity and efficiency of medical services are the concrete manifestation of the high level of development of medical services, and the impact of both on common services is non-linear and the mechanism of action is inconsistent. Specifically, there is an inverted U-shaped non-linear relationship between the level of common prosperity and the efficiency of medical services in China, before the inflection point, the improvement of medical efficiency can promote the level of common prosperity, but after the inflection point, the improvement of medical service efficiency will hinder the realization of common prosperity; the improvement of medical service fairness always has a positive effect on the realization of common prosperity. The improvement of fairness in medical services has always contributed to the realization of common wealth, but it shows a phased characteristic, the stronger the fairness of medical services, the greater the effect on the improvement of the level of common prosperity.

Third, when the degree of government participation is used as the threshold variable, there are single and double threshold effects on the level of equity and efficiency of medical services and the level of common prosperity, respectively. Government participation in the medical service market is an important reason for the inverted U-shaped relationship between medical service efficiency and common prosperity. However, when government participation exceeds 1.4%, the increase in efficiency of health care services hinders the achievement of common prosperity.

The improvement of medical service level can effectively promote the realization of common prosperity, but the relationship between fairness and efficiency of medical service is mutually constrained and mutually promoted. The limited fiscal revenue is an important reason for this constrained relationship, which requires local governments to strive to improve their own financial resources, closely integrate economic development with fiscal development, and pay attention to the construction of the fiscal system, especially in the context of the current macroeconomic growth decline, through the implementation of a series of economic incentive policies, and strive to improve local own financial resources and investment in health care spending.

The government plays a leading role in realizing the equity of medical services, and local governments should clearly position themselves in the process of participating in the medical service market, adhere to the basic function of “covering the bottom line” and “preserving the basics,” and ensure the availability and accessibility of basic medical services in all areas and among the population. The government should break the barriers of the system and mechanism to actively play the demand-led role of the market, encourage and support social forces to provide quality medical services to meet the public's more diversified and multi-level demand for health services.

In the case of government participation, there is uncertainty about the contribution of the level of medical services to the common prosperity. The government's participation should be “localized” and “targeted.” Compared with the eastern region of China, the level of medical services in the central and western regions is still low, and increasing government participation can promote the overall improvement of the level of medical services in the central and western regions; the eastern region should analyze specific problems and not blindly increase medical and health expenditures, and policy making must adhere to the balance between medical equity and efficiency. The government's participation in the medical service market should follow a goal-oriented approach, clarify the government's responsibility in the regional medical business, optimize the structure of financial expenditures, and optimize the structure and management efficiency of the use of funds while increasing the scale of medical expenditures.

## 6. Limitations of the study

The following limitations may exist in the research process of this paper.

1. Most of the sample data in this paper come from the National Statistical Yearbook, and due to the change of statistical caliber of some data and inconsistent statistical time, this paper only selects the statistical data from 2010 to 2019 in the research process. Using only 10 years of data for balanced panel data analysis may lead to bias in the regression results.
2. Common prosperity is a relatively ambitious subject, and although a multidimensional index system is used in this paper to evaluate it, the selection of these indicators may still be insufficient to reflect the true level of common prosperity. The construction of the comprehensive evaluation system on common prosperity still needs further improvement.
3. The government's participation in the level of medical services is multifaceted, including not only financial and taxation support but also the construction of public medical service system, but there is a lack of effective means to measure other means of participation. Therefore, in the empirical analysis of this paper, the indicator of government participation is only measured by the amount of government financial expenditure, which may not be sufficient to truly reflect the degree of government participation.
4. The article only analyzes the situation in China, and the results of the empirical analysis may only be applicable to developing countries similar to China, but may not be applicable to the situation in other countries.

## Data availability statement

The raw data supporting the conclusions of this article will be made available by the authors, without undue reservation.

## Author contributions

BC: conceptualization and writing of the manuscript. XZ: critical review of the manuscript and data analyses. All authors contributed to the article and approved the submitted version.
